# Successful pregnancy and delivery in a patient with chronic myeloid leukemia: a case report and review of the literature

**DOI:** 10.1186/s40064-016-3693-0

**Published:** 2016-12-01

**Authors:** Weiwei Sheng, Naitong Sun

**Affiliations:** Department of Hematology, The Third People’s Hospital of Yancheng, 75 Juchanglu, Tinghu District, Yancheng, 224001 Jiangsu People’s Republic of China

**Keywords:** CML, TKIs, Pregnancy, IFN-α

## Abstract

**Introduction:**

Complications associated with chronic myeloid leukemia (CML) during pregnancy are rare, and management is challenging because very limited data are available on this patient group.

**Case description:**

We herein report a successful pregnancy and delivery in a patient diagnosed with CML. The patient was treated with imatinib (400 mg/day) as a first-line therapy. However, she became pregnant while she was in complete hematological remission and had a complete cytogenetic response. Because she elected to continue the pregnancy to term, imatinib treatment was stopped after 5 months of gestation and the patient was then treated with interferon-alpha for the remainder of her pregnancy. However, the CML did not relapse. She successfully gave birth to a male infant at 39 weeks by cesarean section with no adverse sequelae or malformations.

**Discussion and Evaluation:**

The treatment of pregnant women with CML is difficult because of few available therapeutic options and limited data regarding the potential harm to the fetus. Conception should be planned and TKI therapy discontinued in female patients during pregnancy, and individual risks need to be considered when an unplanned pregnancy occurs.

**Conclusions:**

Our experience will be useful for counseling patients inadvertently exposed to tyrosine kinase inhibitors such as imatinib during pregnancy.

## Background

Chronic myeloid leukemia (CML) accounts for about 15% of cases of adult leukemia, and its median age of onset is about 67 years (O’Brien et al. 2011). Approximately 4870 cases of CML were diagnosed in the US in the year 2010, and 440 of these patients died of CML (Jemal et al. 2010). CML is a hematopoietic stem cell disease caused by a reciprocal translocation between chromosomes 9 and 22, leading to the formation of the Philadelphia chromosome (Ph). The translocation t(9:22) results in the head-to-tail fusion of the breakpoint cluster region (BCR) gene on chromosome 22 at band q11 with the Abelson murine leukemia (ABL) gene located on chromosome 9 at band q34 (Faderl et al. 1999). The product of the fusion gene (BCR/ABL) has been confirmed to play a central role in the development of CML (Daley et al. 1990). The recent use of tyrosine kinase inhibitors (TKIs) in patients with CML has transformed this disease from a fatal condition without aggressive intervention to a chronic condition associated with an extended life expectancy (Hensley and Ford 2003). Therefore, administration of TKIs has become the first-line therapy for patients newly diagnosed with CML. It is rare to encounter a pregnant patient with CML because the median age at the time of diagnosis is approximately 60 years. The main challenge in the use of TKIs to treat pregnant patients with CML is the teratogenic risk for the fetus (Doll et al. 1988). Therefore, although such treatment should be stopped during pregnancy, this may increase the chance of relapse and influence disease progression.

## Case presentation

In June 2003, a 22-year-old woman was diagnosed with a Ph + karyotype and chronic CML. Her physical examination results were normal upon hospital admission. Her peripheral blood test results were as follows: white blood cell count of 227 × 10^9^/L with 2% blasts, 20% myelocytes, 10% metamyelocytes, 12% bands, 53% neutrophils, and 3% lymphocytes; hemoglobin concentration of 79 g/L; and platelet count of 201 × 10^9^/L. Renal and liver test results were normal. The diagnosis of CML was confirmed based on detection of BCR/ABL mRNA transcripts and chromosome banding, which revealed a 46, XY, t(9;22) karyotype. She was first treated with hydroxyurea (2 g/day) and obtained a complete hematologic response (CHR) with no cytogenetic response within 6 months. After 6 months, she was presented to FUNDALEU hospital and began treatment with imatinib (400 mg/day) as a first-line treatment with good tolerance and adherence to the medication. She obtained a major molecular response (MMR) in 12 months. Polymerase chain reaction (PCR) amplification for the BCR/ABL sequence was checked every 6 months to confirm the persistence of the MMR. Although the patient had been advised to use effective methods of contraception during therapy, she reported having had amenorrhea for 2 months in July 2006. Ultrasonography revealed pregnancy at 5 weeks of gestation. Although no data were available to us regarding stopping TKI treatment during pregnancy, after being informed of the possible risks, the patient and her relatives chose to continue imatinib therapy during the first 5 months of gestation. At that point, the imatinib treatment was stopped and the patient was treated with interferon-alpha (IFN-α) at 3 million IU twice a week throughout the remaining pregnancy. All follow-up ultrasound scans during the course of the pregnancy were unremarkable. During pregnancy, the patient maintained the CHR (white blood cell count of <10 × 10^9^/L without splenomegaly); karyotyping and molecular detection were not performed. On 14 February 2007, the patient delivered a healthy male infant at gestational week 39 via cesarean section. The infant weighed 3.2 kg with an Apgar score of 9 at 10 min. Moreover, the child was healthy and developed normally without any evidence of congenital malformations. Two months after delivery, the patient’s peripheral blood counts, mRNA transcripts of BCR/ABL by qualitative PCR, and karyotype were normal. Imatinib treatment (400 mg/day) was restarted to prevent the recurrence of CML. During the next 8 years of follow-up (until the time of this writing), the patient has been adherent to the imatinib therapy and has maintained the CHR and MMR. The child was breast-fed for 1 month, remained healthy, and developed normally without any evidence of congenital malformations. The child’s peripheral blood tests and BCR/ABL mRNA transcripts were assessed every 3 years and showed normal results.

This study was approved by the Ethics Committee of the Third People’s Hospital of Yancheng. Informed written consent was obtained from the patient in accordance with the Declaration of Helsinki.

## Discussion

During the past decade, the clinical view of CML has changed dramatically to a disease requiring lifelong medication that provides substantial disease control, relatively few adverse effects, and nearly normal lifestyles for most patients. In 2001, the first TKI used for the frontline treatment of CML, imatinib, was introduced into the US market, offering durable remission in up to 70% of patients. Dasatinib and nilotinib quickly followed as second-generation TKIs with additional targets.

In general, malignancy during pregnancy is a unique challenge for medical oncologists. When a woman becomes pregnant during chemotherapy or radiotherapy, most cytotoxic agents and radiotherapy have many potential adverse side effects on the fetus (Buekers and Lallas 1998; Zuazu et al. 1991). In addition, many physiological changes that occur during pregnancy (such as an increased plasma volume, a third space created by amniotic fluid, hepatic oxidation, and alterations in renal clearance) can affect drug distribution, metabolism, and excretion (Weisz et al. 2004; Cardonick et al. 2010). Moreover, chemotherapy reportedly inhibited the migration and proliferation of trophoblasts in first trimester human placental explants, which might partially explain the lower birth weights of infants whose mothers received chemotherapy (Matalon et al. 2005).

Overall, malignant diseases are diagnosed in 0.1% of all pregnancies (Slade and James 1991; Rothman et al. 1973). The incidence of leukemia during pregnancy is approximately 1 in 75,000–100,000 pregnancies (Litchtman 1995), most cases of which are acute myeloid leukemia or acute lymphoblastic leukemia. Treatment of CML during pregnancy is difficult because of limited available therapeutic options and the paucity of data regarding potential harm to the fetus. No therapy offered to pregnant women is both completely safe and effective; thus, clinicians are faced with the challenge of balancing the safety of the mother while treating her cancer against the safety of the unborn baby. Termination of pregnancy is considered a safe option for the mother in the first trimester but was not desired in the present case. Because some women with CML are of childbearing age, they often raise issues relating to pregnancy. Therefore, management of the pregnancy should begin from the time of diagnosis of CML, and such patients should be informed of the risks of therapy during pregnancy (Baccarani et al. 2013). At the time of our experience (July 2006), very limited data were available regarding the treatment of women who conceive while undergoing TKI therapy. In March 2006, Ault et al. (2006) reported that three women treated with imatinib had normal pregnancies. The first systematic review of imatinib treatment during pregnancy was reported by Pye et al. (2008), who collected information on 180 women exposed to imatinib during pregnancy among different institutions. The data showed that although most pregnancies in women exposed to imatinib had a successful outcome, exposure was still associated with a risk of serious fetal malformations. Because imatinib inhibits both tyrosine kinases and BCR/ABL expression, it is conceivable that congenital abnormalities might result from the inhibition of members of this extensive enzyme family. Russell et al. (2007) described two pregnancies in women exposed to imatinib during the third trimester. Imatinib was found to be present at high concentrations in the maternal blood and placenta; however, minimal or no drug was detected in the cord blood, suggesting that imatinib and its active metabolite cross the mature placenta only poorly. Since then, many similar cases have been reported, and patients treated with newer TKIs during pregnancy have also been recently described. One patient treated with nilotinib at 400 mg/day until 7.4 weeks of pregnancy delivered an infant with no identified congenital malformations, and the infant had normal development upon follow-up (Conchon et al. 2009). Based on clinical trials and animal experiments, Cortes et al. (2015) reported no apparent risk in the offspring of a small sample of dasatinib-treated men. However, in women treated with dasatinib, risks to the fetus were present in the form of skeletal malformations and detrimental pharmacological effects.

A pregnancy should only be planned after the milestone of a stable complete cytogenetic response or better has been reached from at least 18–24 weeks of gestation. Ultrasonography and planned conception are highly recommended. Therapy should be stopped immediately before or immediately after conception. All TKIs must be avoided during the organogenesis phase (postmenstrual days 31–71, gestational weeks 5–13). PCR detection must be carried out each month or every 2 months to follow the transcript, depending on the molecular biology results (Santorsola and Abruzzese 2015).

If a cytogenetic or hematologic relapse occurs during pregnancy without therapy, the patient should be evaluated on an individual basis with respect to the rapidity of the relapse, the patient’s clinical history, and especially the pregnancy status (weeks of gestation). If necessary, considering the low degree of passage of imatinib and nilotinib into the fetal compartment, imatinib and nilotinib therapy can be considered after the placenta has formed and organogenesis has been completed (Webb and Jafta 2012), while dasatinib therapy should be avoided throughout pregnancy because it can pass to the placenta (Berveiller et al. 2012). Importantly, there is not enough evidence to support this hypothesis, and the use of any TKI at any stage of pregnancy is prohibited according to the manufacturers’ instructions. The risk of using a TKI, even in the third trimester, should be fully discussed with the patient and their partner and might only be justified if the woman is experiencing progression to an advanced phase of CML. In the present case, although the patient conceived accidently and was treated with imatinib for the first 5 months of gestation, the child was closely monitored for more than 8 years and no side effects or abnormal development were found. However, we still believe that effective contraception and planned pregnancy should be suggested during treatment with imatinib. The mother and child in this case are continuing to undergo follow-up.

The use of IFN-α could be an option when stopping TKI therapy in women with a suboptimal response. The maternal IFN-α concentration during pregnancy should be safe for the fetus, and IFN therapy during pregnancy could have the same outcomes as in nonpregnant women (Pons et al. 1995). IFN-α treatment does not significantly increase the risk of major malformations, miscarriage, stillbirth, or preterm delivery above that in the general population (Brojeni et al. 2012). Therefore, treatment with IFN-α during medium-term and terminal gestation in pregnant women with CML is considered safe. IFN-α inhibits cell proliferation through its effects on protein synthesis and RNA breakdown and possibly by immunomodulation. Because of its high molecular weight (19 kDa), it does not cross the placental barrier. However, IFN-α does not inhibit DNA synthesis (Baer et al. 1992). Mutagenicity and teratogenicity have not been observed in animal studies of this agent (Mubarak et al. 2002). Several case reports of IFN-α treatment during pregnancy in women with CML have been published, and no congenital abnormalities in their infants have been reported (Regierer et al. 2006; Al Bahar et al. 2004). Based on our experience, IFN-α therapy appears to be safe in the second and third trimesters of pregnancy. However, whether to use a TKI or IFN-α in the first trimester was difficult to decide because imatinib therapy is not absolutely safe for the fetus (Palani et al. 2015). In this case, when we became aware that imatinib therapy might adversely affect the fetus, IFN-α at 3 million IU twice a week was used throughout the remaining pregnancy. The reassuring follow-up findings during the past 8 years have suggested a good outcome for this treatment.

The latest progress in CML treatment has significantly improved survival and provides most patients with a durable MMR and quality of life. However, the management of CML during pregnancy remains a clinical challenge. We cannot deny that imatinib therapy during pregnancy is associated with an increased risk of spontaneous abortions and serious congenital malformations. Abruzzese et al. (2016) recently presented a complete review on the subject; they suggested that treatment with TKIs has no limitations in male patients trying to conceive, while effective contraception should be encouraged in all female patients because of the risk of fetal complications associated with drug exposure. Conception should be planned and TKI therapy discontinued in female patients during pregnancy, and individual risks need to be considered when an unplanned pregnancy occurs. Women of childbearing age should be advised to apply effective contraceptive measures to avoid conceiving during treatment with TKIs. Women wishing to discontinue treatment to conceive should be counseled and advised of the risk of an adverse response or relapse even if they have achieved a profound and lasting MMR. Molecular monitoring should be carried out at regular intervals throughout pregnancy and consideration given to introducing IFN-α if there is any increase in the tumor load. Overall, our experience will be useful in counseling patients inadvertently exposed to TKIs during pregnancy. The first international register of pregnancy outcomes including details regarding the treatment type (imatinib, other TKIs, and drugs) has been established by the European Leukemia Network. We hope that the treatment of women with CML of childbearing age will be increasingly standardized.

## Conclusions

Although the treatment of CML during pregnancy poses a significant challenge for physicians, and although this particular experience was limited to a single patient, the successful outcome in the present case suggests that the management of pregnancy in patients undergoing treatment with TKIs should be individualized and based on a balance between the relative risks and benefits to the patient and fetus.

## Abbreviations

ABL: Abelson murine leukemia
BCR: breakpoint cluster region
CHR: complete hematologic response
CML: chronic myeloid leukemia
IFN-α: interferon-alpha
MMR: major molecular response
PCR: polymerase chain reaction
Ph: Philadelphia chromosome
TKI: tyrosine kinase inhibitor
